# DephosSite: a machine learning approach for discovering phosphotase-specific dephosphorylation sites

**DOI:** 10.1038/srep23510

**Published:** 2016-03-22

**Authors:** Xiaofeng Wang, Renxiang Yan, Jiangning Song

**Affiliations:** 1 School of Mathematics and Computer Science, Shanxi Normal University, Linfen 041004, China; 2 Institute of Applied Genomics, School of Biological Sciences and Engineering, Fuzhou University, Fuzhou 350002, China; 3 Infection and Immunity Program and The Department of Biochemistry and Molecular Biology, Biomedicine Discovery Institute, Monash University, Clayton, VIC 3800, Australia; 4 Monash Centre for Data Science, Faculty of Information Technology, Monash University, Clayton, VIC 3800, Australia; 5 National Engineering Laboratory for Industrial Enzymes and Key Laboratory of Systems Microbial Biotechnology, Tianjin Institute of Industrial Biotechnology, Chinese Academy of Sciences, Tianjin, 300308, China

## Abstract

Protein dephosphorylation, which is an inverse process of phosphorylation, plays a crucial role in a myriad of cellular processes, including mitotic cycle, proliferation, differentiation, and cell growth. Compared with tyrosine kinase substrate and phosphorylation site prediction, there is a paucity of studies focusing on computational methods of predicting protein tyrosine phosphatase substrates and dephosphorylation sites. In this work, we developed two elegant models for predicting the substrate dephosphorylation sites of three specific phosphatases, namely, PTP1B, SHP-1, and SHP-2. The first predictor is called MGPS-DEPHOS, which is modified from the GPS (Group-based Prediction System) algorithm with an interpretable capability. The second predictor is called CKSAAP-DEPHOS, which is built through the combination of support vector machine (SVM) and the composition of *k*-spaced amino acid pairs (CKSAAP) encoding scheme. Benchmarking experiments using jackknife cross validation and 30 repeats of 5-fold cross validation tests show that MGPS-DEPHOS and CKSAAP-DEPHOS achieved AUC values of 0.921, 0.914 and 0.912, for predicting dephosphorylation sites of the three phosphatases PTP1B, SHP-1, and SHP-2, respectively. Both methods outperformed the previously developed kNN-DEPHOS algorithm. In addition, a web server implementing our algorithms is publicly available at http://genomics.fzu.edu.cn/dephossite/ for the research community.

Protein dephosphorylation is a process that removes the phosphate group from phosphorylated residues in proteins. It is an inverse process of phosphorylation and is catalyzed by phosphatases. Protein dephosphorylation was originally discovered in 1955 by Krebs and Fischer^1^, who were awarded the Nobel Prize in Physiology & Medicine in 1992 for their discovery of reversible phosphorylation. Phosphorylation and dephosphorylation usually occur on neutral but polar residues, such as S (serine), T (Threonine) and Y (tyrosine), and can activate or deactivate all kinds of substrates, including enzymes, structural proteins, signaling molecules, membrane channels, etc. Protein tyrosine phosphatases (PTPs) are known signaling molecules and regulate a myraid of cellular processes, including mitotic cycle, proliferation, differentiation, cell growth, and oncogenic transformation^2^,^3^.

Although high-throughput mass spectrometry (MS) techniques can identify thousands of phosphorylation sites at one time, they cannot detect all phosphorylation sites due to technical limitations. In addition, for a given phosphorylation site, the kinase that catalyzes the phosphorylation cannot be recognized by MS methods. To identify potential phosphorylation substrates for follow-up experimental studies, a variety of novel computational methods have been developed in the last two decades. In a recent review paper of eukaryotic phosphorylation site prediction^4^, 40 methods were present, most of which took into account the sequence information of residues surrounding phosphorylated sites with the assistance of main-stream machine learning approaches, e.g. artificial neural networks, decision trees, genetics algorithms, support vector machines, and conditional random fields, to achieve a reasonable prediction performance. Apart from the sequence information, some methods also took into consideration the structural information, including Phos3D^5^, Predikin 2.0^6^, and NetworKIN^7^.

In contrast, there are highly limited studies that focus on the computational analysis of the inverse process of phosphorylation, dephosphorylation. A manually curated database DEPOD^8^ has been recently constructed to provide the annotations of human phosphatases, their substrates, dephosphorylation sites, involved pathways and external links to small molecule modulators and kinases. Another work on dephosphorylation site prediction was carried out by Wu and his coworkers^9^, who manually collected experimentally validated tyrosine dephosphorylation sites from the literature. They employed the *k*-nearest neighbor (kNN) algorithm to predict potential substrate sites for three protein tyrosine phosphatases, i.e. PTP1B, SHP-1, and SHP-2 which have the majority of dephosphorylation sites in their curated dataset. For the sake of brevity, we termed Wu’s predictor as kNN-DEPHOS in this study.

Protein tyrosine phosphatase 1B (PTP1B) is the first discovered PTP (protein tyrosine phosphatase)^10^ and one of the most studied PTPs. It plays a key role in many biological processes. It has been demonstrated that absence of PTP1B in mice caused enhanced insulin sensitivity and inhibition of PTP1B had potential benefits for diabetes treatment^11^. Both Src homology 2 domain tyrosine phosphatase 1 (SHP-1) and Src homology 2 domain tyrosine phosphatase 2 (SHP-2) contain two tandem SH2 domains. SHP-1 is expressed mainly in hematopoietic cells and plays an important role in hematopoietic cell signaling^12^, while SHP-2 is widely expressed in various tissues and crucial for many cellular functions, such as cell migration, transcription regulation, metabolic control, and mitogenic activation. Previous studies indicate that mutations in its gene may cause acute myeloid Gleukemia and Noonan Syndrome^13^.

The *k*-mer method (substring of protein sequences) has been widely used for extracting features from DNA, RNA, or protein sequences. It has been applied to enhancer identification^14^, microRNA identification^15^, etc. Recently, the discriminative power of this method has been further improved by allowing gaps in *k*-mers^16^. Due to its importance, several elegant web servers or stand-alone tools have been established to generate different modes of *k*-mers for DNA, RNA, and protein sequences, including repDNA^17^, repRNA^18^, and Pse-in-One^19^.

In the present study, we focus on the dephosphorylation site identification for the three phosphatases, PTP1B, SHP-1, and SHP-2. We collected dephosphorylation sites through searching recent literatures and combined these data with the Wu dataset as the training dataset. We then developed two predictors, named as CKSAAP-DEPHOS and MGPS-DEPHOS, which were trained using two different sequence encoding schemes. CKSAAP-DEPHOS is a variant of the *k*-mer method. We performed strict jackknife cross validation and 30 times 5-fold cross validation tests and demonstrated that these two methods provided a competitive performance of dephosphorylation site prediction. Finally, a web server is built and is made freely available at http://genomics.fzu.edu.cn/dephossite/ to the wider research community.

## Methods

### Datasets

In order to train the dephosphorylation site predictor, Wu and his coworkers collected dephosphorylation sites through searching literatures with keywords “protein tyrosine phosphatase^*^ AND dephospho*”^9^. After obtaining the data, they selected the sites of three PTPs, i.e. PTP1B, SHP-1, and SHP-2 that contain the majority of dephosphorylation sites, calculated the amino acid frequencies at residue positions surrounding the dephosphorylation sites, and trained three predictive models for each PTP. Their method was named as kNN-DEPHOS. The Wu dataset contains 57, 47 and 48 dephosphorylation substrate sites for PTP1B, SHP-1 and SHP-2, respectively. We downloaded it from the web server of kNN-DEPHOS (http://cmbi.bjmu.edu.cn/ptpsite/) and used it in our work. To further enlarge the dataset, we searched recent literatures in PubMed by using the key words “dephosphory* AND (ptp1b or shp-1 or shp1 or shp-2 or shp2)” and accordingly obtained 9, 9 and 9 new substrate sites for the three phosphatases, respectively. We then extracted the protein sequences of all substrates from the UniProt database^20^ and identified the dephosphorylation sites by mapping them to the sequences. Peptides of 2*n* + 1 residues that centered around each dephosphorylation site were extracted. Sites located at either N- or C-terminal without sufficient residues for the full window size were complemented with “-”. The extracted peptides constitute the positive training dataset, whereas the (2*n* + 1) residue peptides of all other tyrosine residues in the substrates which have not been experimentally shown to be dephosphorylated constitute the negative training dataset. To remove redundant samples from the initial dataset, the training dataset was further culled to ensure that any two sequences of the 21-residue peptides shared a pairwise sequence identity of less than 70% for both positive and negative samples. A sequence identity threshold of 70% was employed in other previous studies^9^,^21^ and we therefore also used this threshold in this study. After this sequence redundancy reduction procedure, the final training dataset (see Supplementary Dataset 1) contained 63, 50 and 51 positive samples for PTP1B, SHP-1 and SHP-2, and 898, 852, and 718 negative samples for the three phosphates, respectively.

### The MGPS-DEPHOS predictor

In this work, we developed a new dephosphorylation site predictor MGPS-DEPHOS based on the GPS algorithm^22^. The GPS algorithm has been successfully applied to protein post-translational modification site prediction, including phosphorylation^23^, pupylation^24^, calpain cleavage^25^, etc. It consists of four steps: *k*-means clustering, peptide length selection, position weight training and substitution matrix mutation. MGPS-DEPHOS inherited the idea of GPS. However, due to the small size of the positive training dataset, MGPS-DEPHOS omitted the first step *k*-means clustering, where the samples were grouped according to the pairwise peptide similarity. In addition, the fourth step substitution matrix mutation was also omitted by MGPS-DEPHOS, in order to avoid the potential overfitting issue during model training. Therefore, MGPS-DEPHOS comprised of two steps, i.e., peptide length selection and position weight training. Moreover, our implementation of the two steps was different from the original GPS algorithm, which will be described below in detail.

First, for a tyrosine residue in a substrate sequence, its predictive score as a dephosphorylation site is calculated as follows: extract the (2*n* + 1)-residue peptide centering the residue of interest and use the following equation to calculate the similarity score of the peptide with all the positive peptide samples:


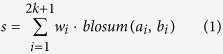


where *w*_*i*_ denotes the weight for the *i-*th position and *blosum*(*a*_*i*_, *b*_*i*_) represents the BLOSUM62 substation score of the two residues at the *i-*th position in the sequences *a* and *b*. Here, the substitution score for position *n* + 1 is 1, i.e. the substitution score for tyrosine to tyrosine in the central position is 1, which allows a more acute tune of the position weights. For those sites that are located near the boundary of the protein sequence, the gap will be represented by the symbol ‘-’, while the substitution score between ‘-’ and ‘-’ or any other residues is assigned as 0. If *s* > 0, the similarity score is *s*, otherwise the similarity score is 0. The final score of the tested peptide would be averaged on all of its similarity scores with all the positive samples in the training set.

To train the position weights in the equation (1), the area under the ROC curve (AUC) of the jackknife cross-validation (see performance measure section) was used to measure the suitability of a group of position weights. We assume that the weight for each position is greater than or equal to 0, except the (*n* + 1)-th position. The detailed training procedure is described as follows:

1. Initialization: set the weight of (*n* + 1)-th position (i.e. the central position), denoted by *w*_*n*+1_, to be 7 (to be in accordance with the fact that the substitution score of Tyr and Tyr in the BLOSUM62 matrix is 7), and the weights of other positions to be 1. Set *s* = 0, which represents whether AUC increases through a round of weight tuning. Set *a* to be the AUC score of the jackknife cross-validation using the initialized position weights. Set position *i* = 1, which is the left end of the peptide;
2. Set *w*_*i*1_ = *w*_*i*_ + 1 and *w*_*i*2_ = *w*_*i*_ − 1, respectively;
3. Substitute *w*_*i*_ with *w*_*i*1_, perform jackknife cross validation, calculate the AUC score and assign it to *a*_1_;
4. If *w*_*i*2_ ≥ 0, substitute *w*_*i*_ with *w*_*i*2_, perform jackknife cross validation, calculate the AUC score and assign it to *a*_2_, otherwise assign *a*_2_ = 0;
5. If *a*_1_ > *a*, then *w*_*i*_ = *w*_*i*1_, *s* = 1, and *a* = *a*_1_. If *a*_2_ > *a*_1_, then *w*_*i*_ = *w*_*i*2_, *s* = 1, and *a* = *a*_2_;
6. Set *i* = *i* + 1. If *i* ≤ 2*n* + 1, go to step 2, otherwise go to step 7;
7. If *s* = 1, set *i* = 1, then go to step 3, otherwise exit.

The original GPS algorithm first selected the best peptide length, and then randomly chose a position to be plus or minus 1 repeatedly, until the value of AUC did not increase. Here, we trained the position weights for peptide lengths ranging from 9 to 61. For each peptide length, the weight for each position was changed one by one in each round, until the AUC did not further increase. Finally, we chose the peptide length that led to the maximal AUC value after position weight tuning. We assume that each position had or hadn’t influence on the classification, thus, except the central position weight, other position weights were restricted to be greater than or equal to 0, which was not restricted by the original GPS algorithm. The reason of such design for MGPS-DEPHOS is explained and discussed in detail in the Results and Discussion section.

### The CKSAAP encoding scheme

The CKSAAP is a popular sequence encoding method and has been recently applied to many prediction problems in bioinformatics^26^,^27^,^28^,^29^ with a competitive performance. CKSAAP is the abbreviation of the composition of *k*-spaced amino acid pairs. In principle, CKSAAP encodes a sequence based on the frequency of amino acid pairs spaced by any *k* (*k* = 0, 1, 2, …) residues. In our work, each dephosphorylation/non-dephosphorylation site was represented by a peptide composed of (2*n* + 1) residues. In the case of *k* = 2, the frequency of amino acid pair “AxxC” (x denotes any residue) is equal to *N*_*AxxC*_/(2*n* − 2), where *N*_*AxxC*_ is the number of the pattern “*AxxC*” in a peptide and 2*n* − 2 is the total number of two-spaced amino acid pairs. Here, we consider “-” as an amino acid type as well, and used *k* = 0, 1, 2, 3 and 4 together to encode a peptide. Thus, the total length of the vector used to encode a peptide is 21 × 21 × 5 = 2205. In this study, we used the CKSAAP encoding scheme to encode each peptide in the training dataset and used the support vector machine (SVM) algorithm to train the CKSAAP-DEPHOS model.

### Support vector machine

SVM is a popular machine learning algorithm and has been widely applied to solve many classification and regression problems in bioinformatics and computational biology^30^,^31^,^32^,^33^,^34^,^35^. Suppose we have a training vector *x*_*i*_ ∈ *R*^*n*^ and the class label *y*_*i*_ = +1 or −1, *i* = 1, 2, …, *l*. To enable the training vectors to be linearly separated, SVM maps them to a higher dimensional feature space using the function *ϕ*(*x*). Then, an optimal hyperplane is constructed to find the training support vectors that are the nearest to the hyperplane of the two classes with the largest margin. SVM requires solving the following optimization problem:


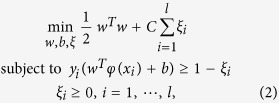


where *ξ*_*i*_ is the slack variable, allowing for misclassifications and *C* > 0 decides the tradeoff between the classification error and the margin. The above optimization problem can be further transformed to its dual problem:


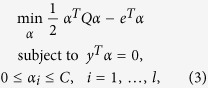


where *e* = [1, ..., 1]^*T*^, *Q* is an *l* × *l* positive semidefinite matrix, 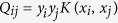 and 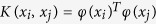, which is called the kernel function. The kernel function implies that we do not need to know the form of the mapping function *φ*(*x*), but only need to create a proper kernel function. The radial basis function (RBF), 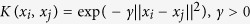, is the most commonly used kernel function. For RBF, its parameter *γ* needs to be finely tuned to obtain an overall best prediction performance. In this paper, the library LibSVM^36^ was employed to implement the SVM algorithm.

### The kNN-DEPHOS algorithm

The kNN-DEPHOS algorithm has been previously used for dephosphorylation site prediction by Wu and his co-workers^9^. First, the similarity scores between the query peptide and all peptides in the training dataset were calculated using the BLOSUM 62 matrix. Due to the dataset imbalance of positive and negative training samples, the similarity scores of positive samples were multiplied by a weight *w*_*i*_. All training samples were then ranked according to the scores from high to low. The final prediction result for the query peptide would be determined by the number of positive and negative training samples ranked within the top *k* samples. In this study, *k* was preliminarily set as 5, which was the same with that in Wu’s method.

### Performance evaluation

The following three methods are often used to examine a predictor for its effectiveness in practical applications: independent dataset test^37^, subsampling test or *k*-fold cross validation test^38^,^39^,^40^,^41^, and jackknife test^42^,^43^. However, considerable arbitrariness exists in the independent dataset test and the *k*-fold cross validation. Only the jackknife test is the least arbitrary that can always yield a unique result for a given benchmark dataset. Therefore, the jackknife test has been widely recognized and increasingly adopted by researchers to examine the quality of various predictors. In *k*-fold cross validation, the training dataset is randomly divided into *k* equally sized subsets. From the *k* subsets, a single subset is taken as the validation set to test the model trained by the other *k*-1 subsets. This procedure is repeated *k* times, until each subset has been used once as the validation set. Jackknife cross validation is a particular type of *k*-fold cross validation, for which the number of subsets is the training set size. Here, we used jackknife test and 5-fold cross validation to assess the performance of different algorithms for predicting dephosophorylation sites. Moreover, 5-fold cross validation performed 30 times to strictly evaluate the prediction performance of each algorithm.

### Performance assessment measures

In this study, four metrics, i.e. sensitivity (SEN), specificity (SPE), Matthews correlation coefficient (MCC), and the area under the ROC curve (AUC) are used to measure the prediction performance of different predictors^44^. The first three metrics are defined as follows:


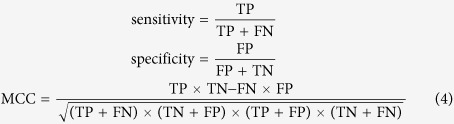


where TP, FP, TN and FN represent the numbers of true positives, false positives, true negatives and false negatives, respectively.

A receiver-operating characteristic (ROC) curve, which plots the true positive rate (sensitivity) against the false positive rate (1-specificity) at different threshold settings, was also used to assess the prediction performance. A predictor with perfect classification has a ROC curve passing through the top left corner (100% sensitivity and 100% specificity). Therefore, the closer the ROC curve is to the top left corner, the better the overall performance of the predictor is. Thus, AUC is used as the primary measure to assess how well a predictor can distinguish between two classes. The R package pROC^45^ was used to plot the ROC curve and calculate the values of AUC in this study.

### Parameter optimization

For many classification algorithms, parameter tuning is crucial to optimize the prediction performance. For kNN-DEPHOS, the peptide length was tuned from 9 to 51 with a step size of 2 and the weight for the positive samples was tuned from 1 to 7 with a step size of 0.1. For MGPS-DEPHOS, the peptide length was tuned from 9 to 61 with a step size of 2 and the position weights were trained using the method described in the “MGPS-DEPHOS predictor” section. When CKSAAP-DEPHOS encoded features were fed into SVM, *γ* and *C* were tuned from 2^−10^ to 2^10^. The parameters for kNN-DEPHOS and MGPS-DEPHOS were trained using jackknife cross validation, while the parameters for CKSAAP-DEPHOS were trained using 30 times of 5-fold cross validation, because it was very time consuming for SVM model training. Finally, the optimal parameters were respectively selected for the three algorithms (listed in Table 1), which led kNN-DEPHOS and CKSAAP-DEPHOS to reach their respective best performance and enabled MGPS-DEPHOS to reach a comparatively better performance.

## Results and Discussions

### Sequence analysis of dephosphorylation peptides

**Figure 1:**
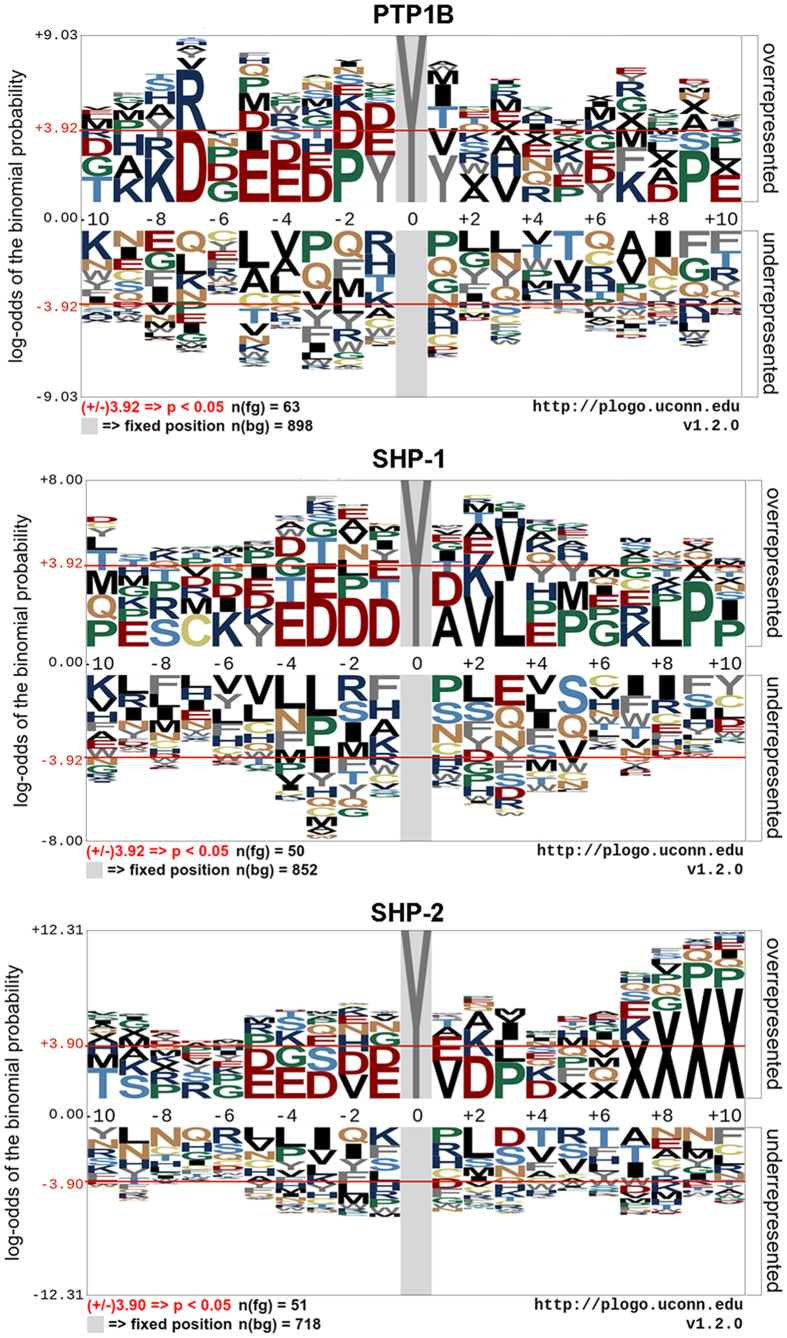
Sequence logo visualization for dephosphorylation sites of PTP1B, SHP-1 and SHP-2. Gap ‘-’ can’t be recognized by pLogo, so it is represented by X in the sequence logo.

In order to investigate the difference of amino acid distributions around the dephosphorylated and non-dephosphorylated tyrosine residues, we computed the 20 amino acid frequencies at each position of the 21-residue peptides of the positive and negative training sets respectively, and employed Fisher’s exact test^46^ to determine the significance of each single amino acid position. Suppose the location of the central tyrosine residue is 0 and the single amino acid position is significant when p-value is less than 0.01, D and R at -7, P at -2 and -9, E at -5, Y at -1 and 1, K at -8 are overrepresented for dephosphorylation peptides of PTP1B. L at 3, P at 9, V at 2 and 3, D at -1, -2, -3, E at -4 are overrepresented for dephosphorylation peptides of SHP-1. ‘-’ at 7, 8, 9 and 10, D at 2, V at 1, P at 3, E at -4 and -1, T at -10 are overrepresented, while L at -4 are underrepresented for dephosphorylation peptides of SHP-2. The more detail of the statistical results could be seen in Supplementary Dataset 2. Furthermore, we submitted the 21-residue training positive and negative peptides to the pLogo web server^47^ (https://plogo.uconn.edu/), and obtained the sequence logo (Fig. 1) for the three phosphatases. In each logo, residue heights are proportional to their statistical significance. Residues along the top of the axis are most overrepresented at each position and residues along the bottom of the axis are most underrepresented. The sequence logos are consistent with the statistical results of Fisher’s exact test.

### Further explanation of MGPS-DEPHOS

The MGPS-DEPHOS algorithm can be considered as a nearest neighbor algorithm because for a test site, its predictive score only takes into account those similarity scores that are greater than 0. Similarity scores can be regarded as the distances between the test site and the positive samples in the training dataset, while 0 is the threshold cutoff. The threshold 0 seems to be fixed, however, when the positional weights are tuned, the similarity scores between the test site and all the positive samples will accordingly change relative to 0, which can be considered as indirect tuning of the threshold of the similarity score. MGPS-DEPHOS only employs positive samples from the training dataset to determine whether a test site is dephosphorylated or not. The position of a residue in a peptide hasn’t or has impact on the prediction. Accordingly, the positional weight was set as 0 or greater than 0 for each position except the central position. In addition, we set the similarity score for the central tyrosine residues to be 1 and in contrast, the weight for the central position could be below 0, thus, the similarity scores can be tuned in any range.

### Prediction performances of different predictors

**Figure 2:**
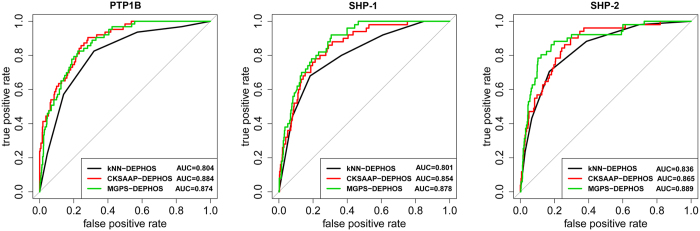
ROC curves of kNN-DEPOHOS, CKSAAP-DEPHOS and MGPS-DEPHOS for the prediction of depohosphorylation sites of the three enzymes, PTP1B, SHP-1, and SHP-2.

To compare the performance of kNN-DEPHOS, MGPS-DEPHOS and CKSAAP-DEPHOS for dephosphorylation site prediction, we performed jackknife cross-validation tests. The ROC curves for these algorithms using the optimal parameters are displayed in Fig. 2. We can clearly see that MGPS-DEPHOS and CKSAAP-DEPHOS performed better than kNN-DEPHOS, for the three respective phosphatases. On the other hand, CKSAAP-DEPHOS performed better than MGPS-DEPHOS on predicting dephosphorylation sites for PTP1B, while GPS-DEPHOS performed better than CKSAAP-DEPHOS for predicting the dephosphorylation sites of SHP-1 and SHP-2.

In order to more objectively compare the performance of different algorithms, we further calculated the values of specificity, sensitivity and MCC at three different specificity levels, i.e. 90% (high), 85% (middle), and 80% (low) and list the results in Tables 2, 3, 4. As the predictive score of kNN-DEPHOS only had six values, its performance at certain specificity levels was not available. We can see that when the specificity was equal to and greater than 80%, CKSAAP-DEPHOS and MGPS-DEPHOS performed comparatively better than the kNN-DEPHOS method.

Furthermore, we performed 30 times of 5-fold cross validation tests to evaluate the prediction performance of the three different algorithms. The average AUC scores are listed in Table 5. Again, these results are consistent with those of the jackknife cross validation tests.

Moreover, we also performed an independent dataset test, where one fifth of the training dataset was selected as the validation set, while the remaining was used to train the model. The results on the independent test indicate the better performance of MGPS-DEPHOS and CKSAAP-DEPHOS. Refer to Table S1 and Figure S1 in the Supplementary File 1 for detailed process and results.

### Performance of the MGPS-DEPHOS algorithm

**Figure 3:**
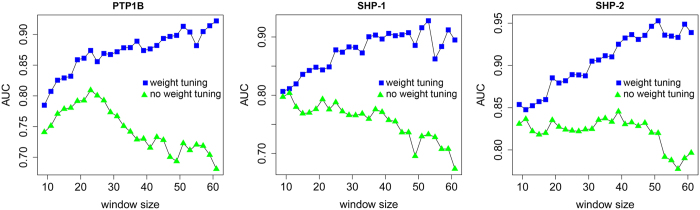
Influence of the peptide length and position weight tuning on the performance of the MGPS-DEPHOS algorithm. Green triangles and blue squares represent AUC values before and after the position weight tuning, respectively.

As discussed above, the MGPS-DEPHOS algorithm included peptide length selection and position weight tuning. Accordingly, we examined the influence of these two factors on the performance of MGPS-DEPHOS. Figure 3 plots the AUC values as a function of the peptide length for the GPS algorithm with and without position weight tuning, respectively. The final position weight obtained by the MGPS-DEPHOS algorithm is locally optimal, rather than globally optimal. Thus, the values of AUC did not always grow with the increase of peptide length, but we can still see that the position weight tuning has a larger impact on the performance improvement. With the increase of the peptide length, the AUC values could become much larger with position weight tuning for the algorithm, but in this work, we employed a reasonably sized peptide length to avoid over-fitting. In addition, the change of AUC values for the algorithm with no position weight tuning could reflect the relative importance of residues at some positions to dephosphorylation site prediction. We can see that the AUC values for PTP1B first grew and then decreased, suggesting that the substrate site of PTP1B mainly depends on the neighboring residues. While for SHP-1 and SHP-2, the AUC values rose up and fell continuously for longer peptide lengths, indicating that residue positions that determine tyrosine dephosphorylation sites for SHP-1 and SHP-2 are more scattered.

As previously discussed, the original GPS algorithm also includes another two steps, *k*-means clustering and matrix mutation. However, in this study, as the size of the positive training set was relatively small, we did not perform the *k*-means clustering as did the original GPS algorithm. Similar to the position weight tuning of the original GPS algorithm, we have also mutated the values of each element in the BLOSUM 62 matrix one by one and observed that the AUC values could reach 1. However, as the positive training dataset is small, this may be caused by giving very small values to comparing reside pairs only appearing in negative peptide and positive peptide comparing, we believe such perfect prediction was achieved due to over-fitting. Therefore, we did not combine this matrix mutation strategy to enhance the prediction in MGPS-DEPHOS.

### The relationship between prediction performance and sequence identity

In order to investigate the potential influence of sequence identity between the training samples on the prediction performance, we further analyzed the pairwise sequence identity between 21-window size peptides. We found that among the 63 substrate sites of PTP1B in the training set, 58 have a sequence identity less than 40% with any other sites, 48 of 50 substrates sites of SHP-1 have a sequence identity of less than 40% with any other sites, while 41 of 47 substrate sites SHP-2 have a sequence identity of less than 40% with any other sites. These results suggest that the sequence identity should have a negelectable effect on the prediction performance.

Furthermore, we also selected positive and negative samples from the training dataset that had a sequence identity of less than 30% with other positive and negative samples, respectively, and subsequently calculated the AUC values using the predictive scores in the jackknife cross validation. The results are listed in Table 6. As can be seen, the AUC values had minor changes compared with those using all samples. This further illustrates that the sequence identity had little influence on the prediction performance.

### The complementarity of three algorithms

If different predictors have a strong complementarity with each other, a proper combination of them may improve the prediction performance. Here, the Pearson’s correlation coefficient (PCC)^48^ of the predictive scores by jackknife cross validation was used to measure the complementarity between any two predictors. The closer of the absolute PCC value for two predictors is to 1, the weaker their complementarity is. As expected, MGPS-DEPHOS and kNN-DEPHOS had a weaker complementarity, with PCCs of 0.582, 0.640 and 0.623, respectively, for the three phosphatase-specific substrate sites. MGPS-DEPHOS and CKSAAP-DEPHOS had a stronger complementarity, with PCCs of 0.338, 0.333 and 0.476, respectively. Therefore, the combination of MGPS-DEPHOS and CKSAAP-DEPHOS might perform better than any individual predictors. Indeed, when using linear SVM to combine the three predictors, we achieved AUC values of 0.926, 0.912 and 0.910 for PTP1B, SHP-1 and SHP-2, respectively. When combining MGPS-DEPHOS and CKSAAP-DEPHOS, we achieved AUC values of 0.921, 0.914 and 0.912, respectively, which were similar results to those of combining the three predictors.

### Identification of potential dephosphorylation substrates of the three PTPs

An use of our developed method is to identify potential dephosphorylation substrates of the three PTPs from large quantities of the human phosphorylation sites^49^. Given the good prediction performance of MGPS-DEPHOS and CKSAAP-DEPHOS, we extracted 36972 human tyrosine phosphorylation sites from phosphorylation site dataset of PhosphoSitePlus^50^, and applied the combination of the two algorithms to scan the sites to identify substrate sites that have not been experimentally demonstrated. Consequently, 3983, 6647 and 8276 phosphorylation sites were identified as dephosphorylation sites of PTP1B at specificities of 90%, 85% and 80% respectively. 4337, 7253 and 10344 phosphorylation sites were identified as dephosphorylation sites of SHP-1 at three specificity levels respectively. 5343, 8608 and 10922 phosphorylation sites were identified as dephosphorylation sites of SHP-1 at three specificity levels respectively.

Two high-confidence dephosphorylation sites, which were not included in the training dataset, were further explored as a case study. Tyr-182 of MAPK14 was predicted to be dephosphorylated by PTP1B at the 90% specificity level. MAPK14 (mitogen-activated protein kinase 14) is a serine/threonine kinase and acts as an essential component of the MAP kinase signal transduction pathway^51^. Experimental results have shown that the phosphorylated MAPK14 on both Thr-180 and Tyr-182 was 10- or 20-fold more active than the phosphorylated MAPK14 only on Thr-180^52^. PTP1B might play a functional role for mediating the activity of MAPK14. Tyr-1165, Tyr-1161 and Tyr-1166 of IGF1R (insulin-like growth factor 1 receptor) were predicted to be dephosphorylated by SHP-1 at the 90% specificity level. IGF1R mediates the function of the insulin-like growth factor 1 and is crucial for tumor transformation and survival of malignant cell^53^. It can be activated by autophosphorylation at Tyr-1165, Tyr-1161 and Tyr-1166^54^. Thus, based on these results, we hypothesize that SHP-1 might be an inhibitor of IGF1R through dephosphorylation at these three sites. The prediction results by MGPS-DEPHOS, CKSAAP-DEPHOS and their combination for the 36972 phosphorylation sites with detailed annotations including protein IDs, site positions and amino acid sequences, are now available at http://genomics.fzu.edu.cn/dephossite. This is a valuable resource for biological experiments.

### Web server

Since user-friendly and publicly accessible web servers are critical for developing practically useful models, simulated methods, or predictors as pointed out and emphasized in some reports^55^,^56^,^57^,^58^,^59^,^60^, a web server for the method presented in this paper was established and is publicly available at http://genomics.fzu.edu.cn/dephossite in order for interested users to perform prediction analysis of potential dephosphorylation substrates and sites. The web server was constructed using the programing languages of Perl, R and HTML. Users are required to submit an amino acid sequence in the FASTA format and designate the substrate site type. Considering that it takes very little time for our server to run the three algorithms (i.e. MGPS-DEPHOS, CKSAAP-DEPHOS and MGPS-DEPHOS + CKSAAP-DEPHOS) and make the prediction, the different prediction results generated by three algorithms will be given together and users actually don’t need to choose the prediction methods. The prediction scores of MGPS-DEPHOS, CKSAAP-DEPHOS and their combination (MGPS-DEPHOS + CKSAAP-DEPHOS) for all tyrosine residues will be generated together by the server and the prediction results according to three specificity levels (90%, 85%, and 80%) will also be returned. In addition, the source code can be downloaded from the server to help users execute the prediction in their local computers and apply our algorithm to other datasets.

## Conclusions

In this work, we have developed two novel effective predictors, namely, MGPS-DEPHOS and CKSAAP-DEPHOS for the prediction of dephosphorylation sites of the three phosphatases, PTP1B, SHP-1, and SHP-2. Originated from the GPS algorithm, MGPS-DEPHOS has a meaningful explanation for its predictive capability. Through strict jackknife cross validation and 30 times of 5-fold cross validation, the two predictors were shown to outperform the previously developed kNN-DEPHOS algorithm. Finally, an online server was built and made freely available at http://genomics.fzu.edu.cn/dephossite. Due to the scarcity of experimentally validated dephosphorylation sites, little attention was paid to computational analysis of dephosphorylation substrates and sites. It is thus our hope that this work can promote the development of this field. We also believe that with the growing of experimentally validated dephosphorylation sites, our algorithm can be well extended to other phosphatases substrate sites prediction and be used as a powerful tool.

## Additional Information

**How to cite this article**: Wang, X. *et al.* DephosSite: a machine learning approach for discovering phosphotase-specific dephosphorylation sites. *Sci. Rep.* **6**, 23510; doi: 10.1038/srep23510 (2016).

## Supplementary Material

Supplementary Information

Supplementary Dataset 1

Supplementary Dataset 2

## Figures and Tables

**Table 1:** Selected parameters for three algorithms.

Method	kNN-DEPHOS		MGPS-DEPHOS		CKSAAP-DEPHOS		
	PL^a^	*w*_*i*_	PL	Positional weight	PL	*γ*	C
PTP1b	23	2.9	23	0,1,3,0,1,0,2,1,2,2,1,-3,3,1,1,1,1,0,2,1,1,1,1	45	2^−7^	2^−3^
SHP1	25	5	25	3,0,1,1,1,0,0,2,1,1,2,1,6,2,2,3,2,2,0,1,0,1,1,0,1	47	2^−9^	2^−2^
SHP2	39	6	25	2,0,0,3,1,0,0,1,1,2,2,2,7,3,3,2,2,0,0,1,1,1,1,1,1	57	2^−10^	2^−2^

**Table 2:** Performance comparison at different specificity levels for PTP1B-specific dephosphorylation site prediction.

Specificity level	High			Middle			Low		
	SPE	SEN	MCC	SPE	SEN	MCC	SPE	SEN	MCC
kNN-DEPHOS	0.955	0.222	0.191	0.860	0.571	0.285	–	–	–
MGPS-DEPHOS	0.900	0.556	0.335	0.850	0.683	0.339	0.800	0.794	0.344
CKSAAP-DEPHOS	0.900	0.619	0.377	0.850	0.667	0.330	0.800	0.746	0.318

“–” denotes that the performance value was not available at the corresponding specificity level.

**Table 3:** Performance comparison at different specificity levels for SHP-1-specific dephosphorylation site prediction.

Specificity level	High			Middle			Low		
	SPE	SEN	MCC	SPE	SEN	MCC	SPE	SEN	MCC
kNN-DEPHOS	0.921	0.44	0.277	–	–	–	0.817	0.68	0.279
MGPS-DEPHOS	0.900	0.600	0.343	0.850	0.700	0.327	0.800	0.780	0.315
CKSAAP-DEPHOS	0.900	0.520	0.293	0.850	0.680	0.316	0.800	0.760	0.304

“–” denotes that the performance value was not available at the corresponding specificity level.

**Table 4:** Performance comparison at different specificity levels for SHP-2-specific dephosphorylation site prediction.

Specificity level	High			Middle			Low		
	SPE	SEN	MCC	SPE	SEN	MCC	SPE	SEN	MCC
kNN-DEPHOS	0.933	0.431	0.315	0.830	0.706	0.330	–	–	–
MGPS-DEPHOS	0.900	0.745	0.458	0.850	0.804	0.411	0.800	0.882	0.394
CKSAAP-DEPHOS	0.900	0.569	0.345	0.850	0.647	0.319	0.801	0.745	0.320

“–” denotes that the performance value was not available at the corresponding specificity level.

**Table 5:** Average AUC on 5-fold cross validation.

Method	PTP1B	SHP-1	SHP-2
kNN-DEPHOS	0.785	0.795	0.825
MGPS-DEPHOS	0.853	0.863	0.877
CKSAAP-DEPHOS	0.873	0.841	0.866

**Table 6:** The predictive performance between different methods for the prediction of dephosphorylation sites of the three phosphatases, PTP1B, SHP-1, and SHP-2.

Method	PTP1B (42, 291)	SHP-1 (41, 301)	SHP-2 (37, 298)
kNN-DEPHOS	0.725	0.765	0.806
GPS-DEPHOS	0.84	0.881	0.881
CKSAAP-DEPHOS	0.894	0.848	0.873

The two numbers in the parentheses indicate the size of the positive and negative data set.
