# Supplementary material for: DephosSite: a machine learning approach for discovering phosphotase-specific dephosphorylation sites
Source: Sci Rep. 2016 Mar 22;6:23510. doi: 10.1038/srep23510 (PMC4802303; doi:10.1038/srep23510)
Supplement: Supplementary Information [file srep23510-s1.doc]

**Supplementary Information**

**DephosSite: a machine learning approach for discovering phosphotase-specific dephosphorylation sites**

**Xiaofeng Wang^a^, Renxiang Yan ^b^, Jiangning Song^c,d,e,*^**

*^a^**School of Mathematics and Computer Science, Shanxi Normal University, Linfen 041004, China*

*^b^Institute of Applied Genomics, School of Biological Sciences and Engineering, Fuzhou University, Fuzhou 350002, China*

*^c^Infection and Immunity Program and The Department of Biochemistry and Molecular Biology, Biomedicine Discovery Institute, Monash University, Clayton, VIC 3800, Australia*

*^d^Monash Centre for Data Science, Faculty of Information Technology, Monash University, Clayton, VIC 3800, Australia*

*^e^National Engineering Laboratory for Industrial Enzymes and Key Laboratory of Systems Microbial Biotechnology, Tianjin Institute of Industrial Biotechnology, Chinese Academy of Sciences, Tianjin, 300308, China*

*Corresponding author: [Jiangning.Song@monash.edu](mailto:Jiangning.Song@monash.edu)

**Independent dataset test**

In order to perform the independent test, we randomly selected one fifth of the training dataset described in the manuscript as an independent dataset. The remaining four fifths were used to re-train the model. The model parameters were selected through cross validation as described in the manuscript and are displayed in Table S1. The trained models were further used to predict the dephosphorylation sites in the independent test dataset. The corresponding ROC curves and AUC values for the three different algorithms are displayed in Figure S1 below.

**Table S1. The prediction performance on the independent test dataset and the selected parameters of the three algorithms.** PL denotes the peptide length.

| Method | kNN-DEPHOS | | MGPS-DEPHOS | | CKSAAP-DEPHOS | | |
| --- | --- | --- | --- | --- | --- | --- | --- |
|  | PL | *w_i_* | PL | Positional weight | PL | *γ* | C |
| PTP1B | 29 | 2.3 | 27 | 3,0,0,1,1,1,1,0,2,0,1,2,2,4,2,1,3,0,1,2,1,0,1,0,1,1,0 | 45 | 2^-9^ | 0.25 |
| SHP1 | 23 | 4.0 | 29 | 1,1,2,0,1,0,1,0,0,2,1,1,1,1,7,2,2,2,2,2,0,1,0,1,1,0,1,0,1 | 45 | 2^-8^ | 0.125 |
| SHP2 | 47 | 5.3 | 23 | 0,0,2,1,0,0,1,1,2,2,1,4,2,2,1,1,0,0,1,1,1,1,1 | 57 | 2^-10^ | 0.125 |


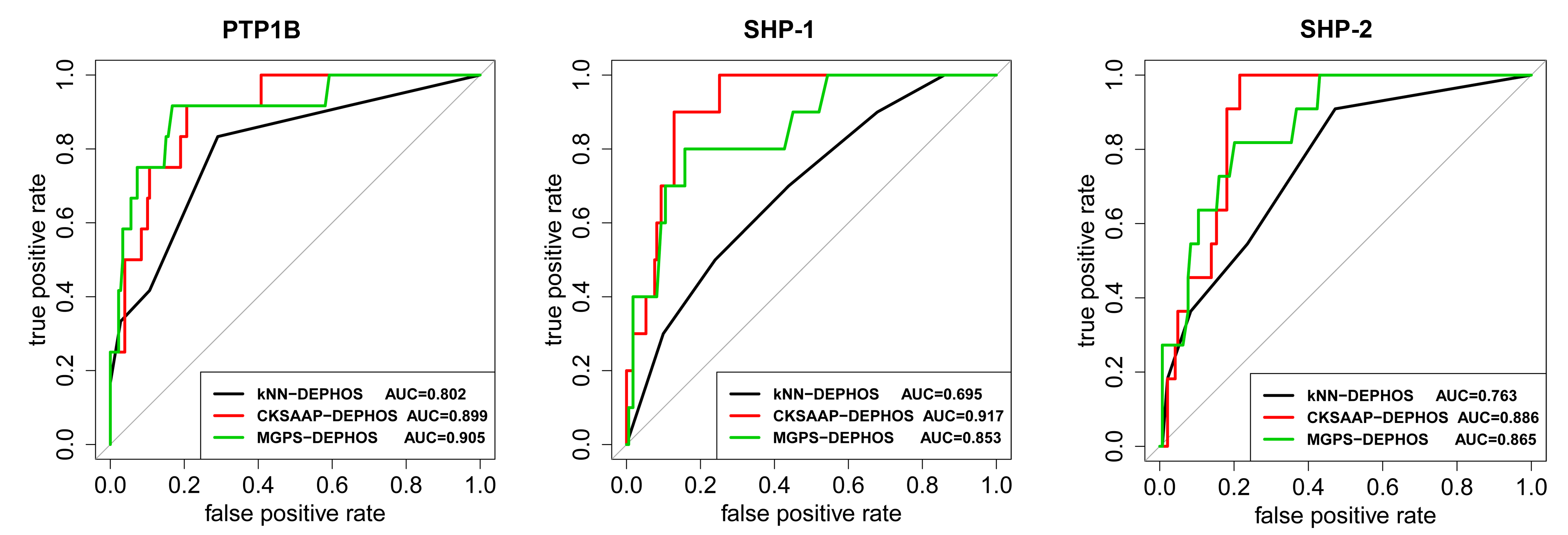


**Figure S1. ROC curves of kNN-DEPOHOS, CKSAAP-DEPHOS and MGPS-DEPHOS on the independent dataset test of the three enzymes, PTP1B, SHP-1, and SHP-2.**
